# Supplementary material for: Complexome profiling of the Chlamydomonas psb28 mutant reveals TEF5 as an early PSII assembly factor
Source: Plant Cell. 2025 Mar 17;37(6):koaf055. doi: 10.1093/plcell/koaf055 (PMC12164590; doi:10.1093/plcell/koaf055)
Supplement: koaf055_Supplementary_Data [file koaf055_supplementary_data.zip › psb28_interactive.zip!/plots/intensity/qualityPlots/peptides/petA_Cre-1.g2716989.t1.p1.html]

### Cre-1.g2716989.t1.p1

Trivial name: petA  
SeqCoverage [median]: 64.70  
Mass: 34.29 kDa  
  
 

| Sequence | MissedCleavage | Mass [Da] | wtIntensity | muIntensity | Score |
| --- | --- | --- | --- | --- | --- |
| ANGEVVVDK | 0 | 929.48 | 3651.73 | 15147.95 | 172.94 |
| ANGEVVVDKIPAGPDLIVK | 1 | 1933.09 | 188115.15 | 689610.41 | 201.18 |
| EGQTVQADQPLTNNPNVGGFGQAETEIVLQNPAR | 0 | 3591.76 | 2197.47 | 23675.31 | 126.05 |
| EKVGNLYYQPYSPEQK | 1 | 1941.95 | 22595.52 | 17407.26 | 134.88 |
| GDLNVGMVLILPEGFELAPPDR | 0 | 2351.22 | 2095.53 | 0.00 | 121.47 |
| GDLNVGMVLILPEGFELAPPDRVPAEIK | 1 | 2988.60 | 7696.36 | 0.00 | 100.23 |
| GDLNVGMVLILPEGFELAPPDRVPAEIKEK | 2 | 3245.74 | 14866.18 | 6933.98 | 111.82 |
| GGFEVSIEK | 0 | 964.49 | 89991.68 | 182207.17 | 208.37 |
| GQVYPDGK | 0 | 862.42 | 152749.10 | 146436.81 | 137.18 |
| GQVYPDGKK | 1 | 990.51 | 65180.31 | 100631.96 | 163.15 |
| GRGQVYPDGK | 1 | 1075.54 | 23930.28 | 37368.66 | 67.33 |
| GRGQVYPDGKK | 2 | 1203.64 | 33211.46 | 40716.32 | 96.95 |
| IPAGPDLIVK | 0 | 1021.62 | 61705.37 | 143415.31 | 149.94 |
| IVAITALSEK | 0 | 1043.62 | 1047960.75 | 1894195.53 | 177.30 |
| IVAITALSEKK | 1 | 1171.72 | 74624.15 | 189065.04 | 166.86 |
| IVAITALSEKKGGFEVSIEK | 2 | 2118.19 | 0.00 | 14179.25 | 63.34 |
| KGDLNVGMVLILPEGFELAPPDR | 1 | 2479.31 | 12354.91 | 0.00 | 115.84 |
| KGDLNVGMVLILPEGFELAPPDRVPAEIK | 2 | 3116.69 | 37014.33 | 0.00 | 120.83 |
| KGDLNVGMVLILPEGFELAPPDRVPAEIKEK | 3 | 3373.83 | 2858.68 | 15229.18 | 73.49 |
| KGGFEVSIEK | 1 | 1092.58 | 384735.47 | 486396.13 | 236.35 |
| KSNNTIYNASAAGK | 1 | 1437.72 | 118085.80 | 137582.63 | 196.84 |
| KSNNTIYNASAAGKIVAITALSEK | 2 | 2463.33 | 1628.40 | 20860.20 | 82.59 |
| KSNNTIYNASAAGKIVAITALSEKK | 3 | 2591.43 | 0.00 | 6319.98 | 71.09 |
| KYSEMVVPILSPDPAK | 1 | 1772.94 | 78885.81 | 239056.14 | 133.28 |
| KYSEMVVPILSPDPAKNK | 2 | 2015.08 | 18093.17 | 6448.34 | 92.31 |
| NILVVGPVPGK | 0 | 1091.67 | 304275.74 | 940447.29 | 209.58 |
| NILVVGPVPGKK | 1 | 1219.77 | 755557.99 | 1000879.40 | 158.76 |
| NKNVSYLK | 1 | 964.53 | 14903.75 | 13990.53 | 133.86 |
| NKNVSYLKYPIYFGGNR | 2 | 2032.05 | 692.74 | 37435.76 | 60.47 |
| NVSYLK | 0 | 722.40 | 120872.19 | 77711.48 | 107.22 |
| NVSYLKYPIYFGGNR | 1 | 1789.92 | 68382.31 | 606225.15 | 193.45 |
| NVSYLKYPIYFGGNRGR | 2 | 2003.04 | 0.00 | 2199.63 | 41.97 |
| QFEKVQLAEMNF | 1 | 1482.72 | 1675.26 | 9565.64 | 205.56 |
| QVLANGK | 0 | 728.42 | 32602.02 | 66164.07 | 149.06 |
| SNNTIYNASAAGK | 0 | 1309.63 | 298759.20 | 368876.43 | 292.08 |
| SNNTIYNASAAGKIVAITALSEK | 1 | 2335.24 | 2104.70 | 7588.67 | 113.73 |
| SNNTIYNASAAGKIVAITALSEKK | 2 | 2463.33 | 951.07 | 6152.60 | 67.61 |
| VGNLYYQPYSPEQK | 0 | 1684.81 | 408107.16 | 932791.50 | 289.22 |
| VGNLYYQPYSPEQKNILVVGPVPGK | 1 | 2758.47 | 0.00 | 8782.34 | 106.35 |
| VGNLYYQPYSPEQKNILVVGPVPGKK | 2 | 2886.56 | 1046.99 | 28354.74 | 85.70 |
| VPAEIKEK | 1 | 912.53 | 5665.98 | 9349.35 | 162.25 |
| VQLAEMNF | 0 | 950.45 | 414882.74 | 862596.25 | 212.20 |
| YPIYFGGNR | 0 | 1085.53 | 455550.92 | 951457.18 | 191.91 |
| YPIYFGGNRGR | 1 | 1298.65 | 0.00 | 1419.55 | 67.56 |
| YSEMVVPILSPDPAK | 0 | 1644.84 | 471380.66 | 1115215.38 | 240.00 |
| YSEMVVPILSPDPAKNK | 1 | 1886.98 | 129380.17 | 211300.60 | 211.09 |
|  |  |  | 5931019.2 | 11671387.1 |  |
